# Supplementary material for: Soldier Caste-Specific Protein 1 Is Involved in Soldier Differentiation in Termite Reticulitermes aculabialis
Source: Insects. 2022 May 26;13(6):502. doi: 10.3390/insects13060502 (PMC9224846; doi:10.3390/insects13060502)
Supplement: Supplementary file 1 [file insects-13-00502-s001.zip › insects-1736552-supplementary.pdf]

Supplementary table S1

genes	Thesequence of Primer (5'-3')
RaSsP1	ACTGTGCTTGGCGCTGTC<br>CTGGGATGTGGTATTGCTTT
Met	GCCCTCATCATCCGCCTT<br>CTTGCCATCACGAGAACG
Kr-h1	AGCAGCCCAGATTTACCT<br>GTCTTCGCCCTCCTTTCC
Br-C	GCACAGACAGTTGGGAGA<br>CAACACCTTGGTTTGAGT
RPL13a	TCTGTGGAGGACGGTTAG<br>ACTTTCTGCCTGGTTTCA
EF1-alfa	CCCTTCGTCTTCCTCTTC<br>CTCCAGCGACATAACCAG
